# Artemisinin and its derivatives: all-rounders that may prevent the progression from lung injury to lung cancer

**DOI:** 10.3389/fphar.2025.1602581

**Published:** 2025-11-19

**Authors:** Xin Xie, Qian Chen, Cui Guo, Jinhao Zeng, Qingying He

**Affiliations:** 1 The Second Affiliated Hospital of Chongqing Medical University, Chongqing, China; 2 Hospital of Chengdu University of Traditional Chinese Medicine, Chengdu, China; 3 Jiangxi University of Chinese Medicine, Nanchang, China

**Keywords:** artemisinin, artemisinin derivatives, lung disorders, lung cancer, multi-step progression

## Abstract

Lung cancer is the major cause of cancer-related deaths worldwide and may occur as a multistep progression. Lung disorders, such as pneumonia and lung injury (Phase Ⅰ), induce inflammatory responses, activate fibroblasts, leading to collagen deposition and the formation of fibrotic lesions. Pulmonary fibrosis (PF) and chronic obstructive pulmonary disease (COPD) (Phase Ⅱ), further induce endoplasmic reticulum stress and DNA damage, leading to cellular mutations that increase the risk of cancer and promote lung cancer (Phase Ⅲ). Based on the fact that disease progression is a progressive and dynamic process, new drugs are urgently required to prevent the progression of lung diseases to cancer. Artemisinin and its derivatives have anti-viral, anti-inflammatory, anti-fibrotic, immunoregulatory, and anti-cancer activities. Hence, we reviewed the multi-step actions of artemisinin and its derivatives on the trilogy from lung diseases to lung cancer, and investigated the underlying mechanism involved. Substantially, actions of anti-inflammation, oxidative stress and apoptosis produced by artemisinin and its derivatives were found throughout the three phases, and NF-κB, Keap1/Nrf2 and PI3K/Akt may be the key signaling pathways. Specifically, in phase of inflammation and injury (phase Ⅰ), artesunate, dihydroartemisinin, and artemether alleviate the symptoms of pneumonia and lung injury by regulating inflammatory responses, oxidative stress, apoptosis, and endoplasmic reticulum stress. In the precursor phase (phase Ⅱ), artesunate and dihydroartemisinin exert antifibrotic and antimycobacterial properties and ameliorate PF and COPD by inhibiting inflammation, modulating oxidative stress, and decreasing cell proliferation. In the cancer phase (phase Ⅲ), artemisinin, artesunate, and dihydroartemisinin could modulate glycolysis, promote apoptosis, ferroptosis, and autophagy, inhibit cell proliferation, invasion, and angiogenesis, and alleviate radiation resistance to exert their anticancer effects. Additionally, current research is focused on nanoscale delivery systems to increase the bioavailability and improve drug stability, to enhance the therapeutic efficacy of these compounds. Collectively, artemisinin and its derivatives are the potential clinically useful therapeutic agents for protecting lungs and hampering the dynamic development processes of lung diseases to lung cancer.

## Graphical Abstract

**Figure:**
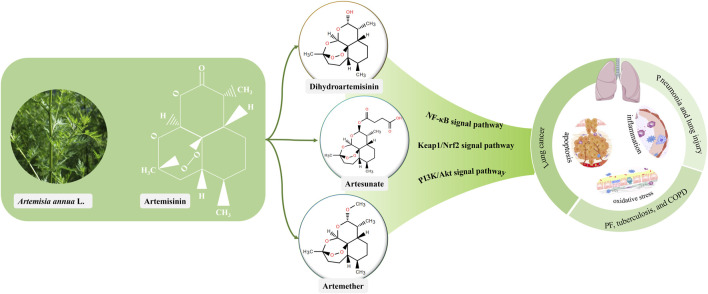

## 1 Introduction

Lung cancer is the leading cause of cancer-related mortality globally with an estimated 80,000 deaths annually. The mortality from lung cancer is increasing as the population grows and ages (Kocarnik et al., 2022; Fan et al., 2023; Sun et al., 2023; Sung et al., 2021). The incidence and mortality rates are higher in developing countries than in developed countries, exacerbating global disease burden and economic strain (Apple et al., 2023).

Normal lung cells become malignant due to genetic mutations induced by several factors, such as environmental and genetic factors. These mutations occur in genes that regulate cell cycle, DNA repair, and angiogenesis and favor cell growth and survival, resulting in abnormal cell growth and division, unlimited proliferation and spreading of cells (by overriding the normal cell cycle regulation and evading programmed cell death), and tumor formation (Long et al., 2019; Baykara et al., 2015). The transformation from a normal to malignant phenotype involves key alterations, such as inactivation of tumor suppressor genes, activation of proto-oncogenes, dysregulation of apoptosis and telomerase control, sustained angiogenesis, and tissue invasion (Breuer et al., 2005). Fortunately, the occurrence and development of lung cancer is a multistep process, early detection of cancer at the pre-invasive stage may provide an opportunity to inhibit or slowdown the progression of malignant disease, ultimately improving the prognosis in patients (Lantuéjoul et al., 2009; Mascaux, 2008).

The World Health Organization Classification specifies lung cancer precancerous lesions as squamous epithelial atypia and carcinoma *in situ*, atypical adenomatous hyperplasia, and infiltrative idiopathic pulmonary neuroendocrine cell hyperplasia, which are the early warning signals for the development of lung cancer (Kerr, 2001). Although only 15%–20% of idiopathic pulmonary fibrosis (IPF) cases will progress to lung cancer (Yao et al., 2025), pulmonary diseases such as pneumonia, lung injury, PF, tuberculosis, and COPD are considered risk factors for lung cancer (Li C. et al., 2022; Bhat et al., 2022; Wang L. et al., 2023). The “inflammation-fibrosis-cancer” cascade is not an inevitable pathway, but it does create a favorable environment for tumor development (Feng et al., 2025). In other words, people with pulmonary diseases are at an increased risk of developing lung cancer (Brenner et al., 2012; Brenner et al., 2011).

Pneumonia is mainly treated with antibiotics to eliminate microorganisms. PF has no specific drug, and slowing down the progression of the disease to alleviate the symptoms is the main goal of treatment. The treatment for tuberculosis includes the long-term use of anti-tuberculosis drugs (Khan et al., 2021; Nguyen et al., 2022; Min et al., 2022). COPD is treated with inhaled medications (e.g., bronchodilators and steroids) to ameliorate symptoms, and smoking cessation along with a pulmonary rehabilitation program are recommended to improve lung function (Gupta et al., 2019). Common treatment options for lung cancer include surgical resection, radiotherapy, chemotherapy, targeted therapy, and immunotherapy (Sowa et al., 2017). Collectively, these treatment approaches focus on eliminating infection, immunomodulation, mechanical ventilation, and removal of malignant tissue (surgical interventions). Unquestionably, innovative drugs which can treat lung diseases and inhibit their dynamic development progression to lung cancer are attractive.

Universally acknowledged that artemisinin and its derivatives are the most effective drugs for treating drug-resistant malaria and have a fast-acting and low-toxicity profile. Artemisinin is derived from the plant *Artemisia annua* L., and its common derivatives include artesunate, dihydroartemisinin, artemether (Posadino et al., 2023; Tang et al., 2023). Evidence is mounting that artemisinin and its derivatives can reduce the disease burden of lung cancer and inhibit the progression of lung diseases to lung cancer. Here, we focused on the multistep pathogenesis of lung cancer, discussed the progression from pneumonia to lung cancer, and investigated the regulatory effect of artemisinin and its derivatives on each step of cancer progression. In addition, toxic side effects of drugs and delivery routes of artemisinin and its derivatives were reviewed.

## 2 Lung injury to lung cancer: a multistep dynamic development process

The lungs are the central organs in the human respiratory system, carrying several important functions, such as respiratory regulation, immune function, and pulmonary circulation (Schneider et al., 2021). The success or failure of pulmonary defense mechanisms determines the emergence of clinical diseases. Pulmonary defense is dependent on the immune and nervous systems. Immune defense is the ability of cells (such as neutrophils and macrophages) and molecules in the lungs to clear pathogens from the alveoli and prevent them from entering the bloodstream (Hiroki et al., 2021). Neurological defenses include aerodynamic filtration, ciliary motility, and other forces that detect external threats through sensory neurons and drive the movement of respiratory fluids (Green et al., 1977). The disruption of these defense mechanisms by various factors leads to lung diseases, which can increase the risk of lung cancer (Shehata et al., 2023; Zou et al., 2022; Cha et al., 2023; Hamada et al., 2023; Hu et al., 2021; Lu et al., 2022; Zhu et al., 2020). It is evident that multiple lung diseases are interconnected, and the evolving transition from lung injury to lung cancer is a dynamic development process.

Pneumonia is a lung infection caused by bacteria, viruses, or fungi (Michelet et al., 2021). Microbial pathogens enter the respiratory tract, triggering an inflammatory response that damages lung tissue (Tian et al., 2023). In addition, inflammatory response increases the permeability of the alveolar walls, leading to the leakage of fluid and cells from the alveoli and the formation of parenchymal lung lesions (Holloway et al., 2018). The uncontrolled inflammatory responses may aggravate lung infections and cause serious lung damage (Garg et al., 2021). Hence, lung injury is a more serious disorder compared with pneumonia. In addition to infection and inflammation, lung injury is usually caused by trauma and several other factors (Habet et al., 2023). The inflammatory response is further exacerbated in lung injury, leading to diffuse damage to alveolar epithelial cells, decrease in lung surface-active substances, destruction of the alveolar walls, increased permeability of the basolateral membranes, accumulation of intra-alveolar fluid, accumulation of polymorphonuclear leukocytes, parenchymal cell damage, and interstitial edema (Meng et al., 2018). Ultimately, continued damage to the lung epithelium due to uncontrolled inflammation leads to abnormal lung tissue repair. Moreover, inflammatory response activates fibroblasts, leading to increased collagen synthesis. This collagen is deposited in the lung tissue to form fibrotic lesions (Ammar et al., 2019; Wei et al., 2023). Accordingly, we refer to pneumonia and lung injury as the phase Ⅰ of dynamic development processes of lung diseases to lung cancer.

PF involves fibrotic tissue proliferation in the lungs. Its pathologic progression involves complex interactions between epithelial cells, mesenchymal stem cells, fibroblasts, immune cells, and endothelial cells (Zhang and Wang, 2023). Fibrosis, overgrowth, sclerosis, and scarring of various tissues are attributed to the expansion of activated mesenchymal stromal cells (myofibroblasts), leading to excessive deposition of extracellular matrix components in basement membranes and interstitial tissues (Makena et al., 2023). Gradual loss of elasticity and function of lung tissue occurs as the fibrous tissue proliferates and deposits, leading to dyspnea and other clinical symptoms (Marts et al., 2019). Molecular and cellular processes, such as myofibroblast/mesenchymal transition, myofibroblast activation and uncontrolled proliferation, endoplasmic reticulum stress, altered expression of growth factors, and oxidative stress, link PF to lung cancer and increase the risk of cancer development by 7%–20% (Ballester et al., 2019).

In addition, lung damage and fibrosis are frequently observed in tuberculosis (Ravimohan et al., 2018). It is a chronic infectious disease caused by *Mycobacterium tuberculosis*, which enters the lungs and triggers an immune response that results in the formation of tuberculous nodules (Huang et al., 2022). These nodules contain macrophages and lymphocytes that control the spread of bacilli (Fallahi-Sichani et al., 2010). However, these nodules develop into foci if the immune response fails to control the infection (Khan et al., 2019). Tuberculosis is a known risk factor for lung cancer because chronic inflammation and fibrosis may induce genetic mutations and DNA damage, leading to lung cancer (Schabath and Cote, 2019; Hwang et al., 2022).

COPD is a chronic inflammatory disease characterized by airway obstruction, alveolar destruction, and reduced lung function. The disease is projected to become the third leading cause of death worldwide by 2030 (Li et al., 2023). Patients with COPD have a 4–6 times higher risk of lung cancer compared with non-COPD patients (Shih et al., 2021). Smoking is the most common cause of COPD and lung cancer, and approximately 85%–90% of cases are associated with exposure to tobacco smoke (Czarnecka-Chrebelska et al., 2023). Harmful substances in tobacco smoke trigger an inflammatory response in the airways, leading to increased chemotaxis of bronchial mucosal cuprocytes and mucus secretion. The subsequent release of inflammatory cells can damage lung tissue leading to PF, destruction of the alveolar walls, and reduced lung function (Cheng et al., 2015). In addition, inflammation can increase DNA damage and mutations, leading to tumor proliferation, anti-apoptotic effects, angiogenesis, invasion, and metastasis (Zhou et al., 2023). In the continuous progression of pneumonia and lung injury, various lung diseases (PF, tuberculosis and COPD) emerge, all of which can directly raise the risk of lung cancer. Thus, we define them as the phase Ⅱ of the dynamic development processes.

Lung cancer is a malignant tumor segregated into two main groups, namely non-small cell lung cancer (NSCLC) and small-cell lung cancer (SCLC), with NSCLC accounting for 80%–85% of the diagnosed cases (Guo et al., 2021). Inflammation, immunity, oxidative stress, cell proliferation, apoptosis, and mitochondrial dysfunction play important roles in the development and progression of lung cancer (Liu J. et al., 2023; Wang et al., 2024a; Pang et al., 2024). Inflammation can damage lung tissue and induce DNA damage in lung cells, mitochondrial DNA damage leads to the dysregulation of mitochondrial quality control, which lays the foundation for the malignant transformation from stage II (such as pulmonary fibrosis) to stage III (lung cancer), thereby increasing the risk of lung cancer (Taucher et al., 2022). Under normal circumstances, immune cells can inhibit the development of lung cancer by recognizing and removing abnormal lung cells. In contrast, tumor cells often evade immune surveillance through various “immune escape” mechanisms to avoid recognition and elimination, thereby promoting tumor growth and metastasis (Rui et al., 2023). In addition, oxidative stress, mitochondrial quality control and mechanisms involved in cell proliferation, apoptosis, and cell cycle regulation are closely related to the occurrence and development of lung cancer (Deng et al., 2013; Larson-Casey et al., 2020; Chang et al., 2023). Lung cancer represents the final stage of the development processes and is classified as phase Ⅲ.

**FIGURE 1:**
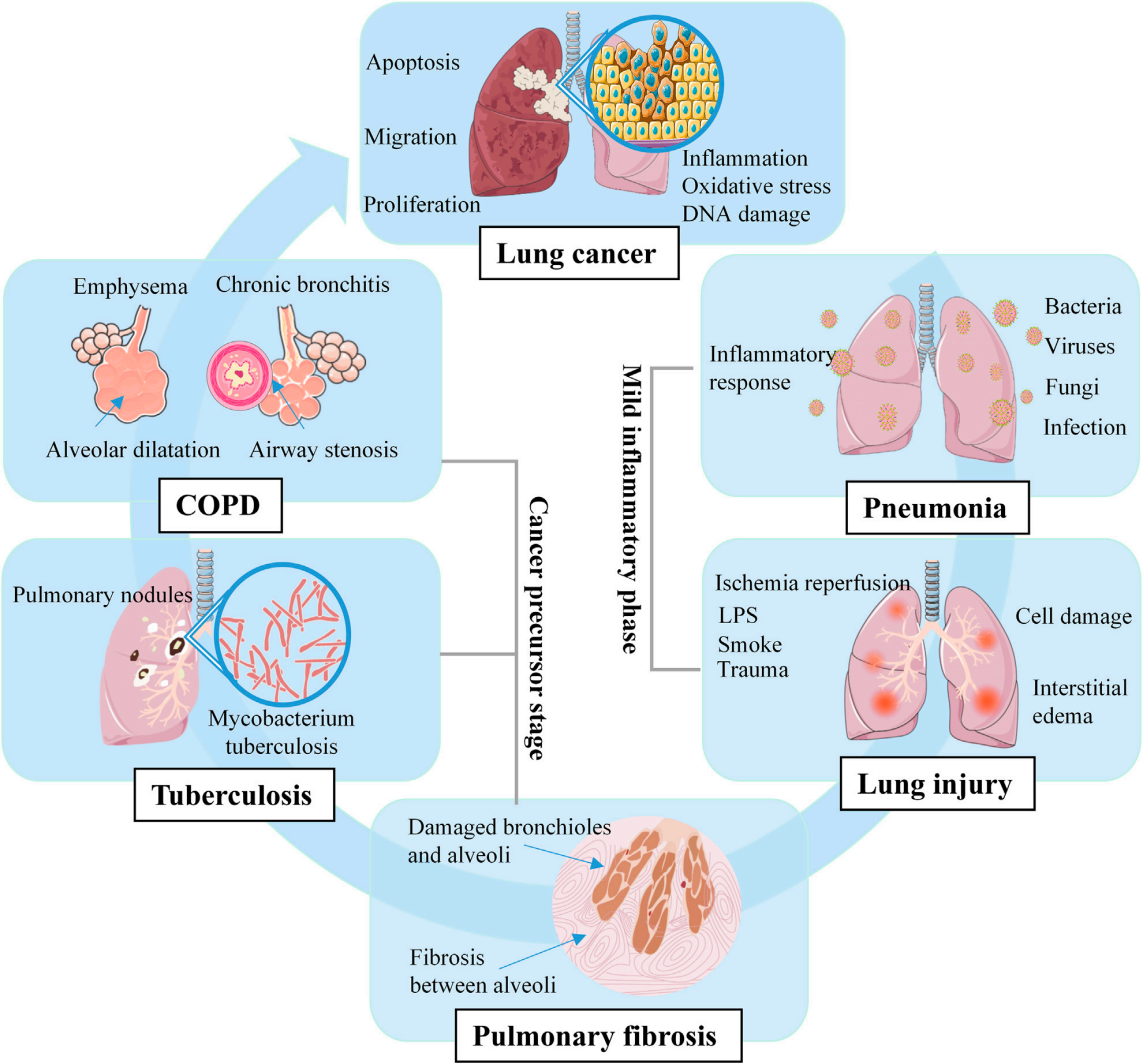
Multistep pathogenesis of lung cancer.

Overall, prior lung disease increases the risk of lung cancer (Brenner et al., 2012; Brenner et al., 2011). Lung cancer is a multistep and multidimensional process, pneumonia and lung injury are inflammatory diseases that increase the risk of lung cancer, PF, tuberculosis, and COPD are precursor diseases that further promote the development of lung cancer (Figure 1). Compared with treating cancer, the approach of prevent is more feasible and practical, identifying risk factors and implementing prevention strategies are key to reduce the global burden of lung cancer (Garrison et al., 2021).

## 3 Artemisinin and its derivatives

**FIGURE 2:**
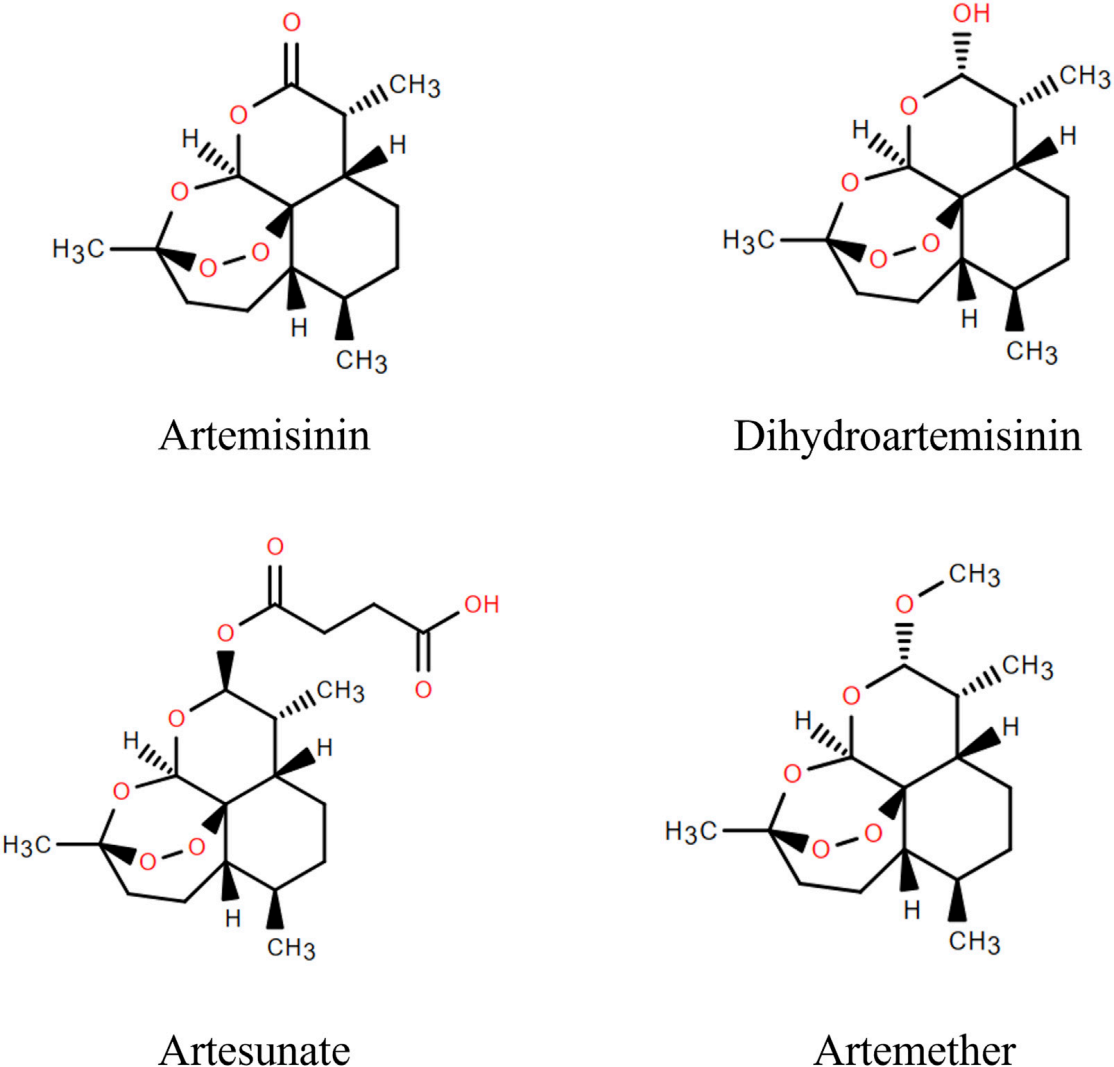
Artemisinin and its derivatives.

Artemisinin, a sesquiterpene lactone, is a first-line drug for the treatment of malaria. It was first extracted from *A. annua* L. by Tu Youyou and colleagues in 1972 (Fikadu and Ashenafi, 2023), and is a colourless crystal with the molecular formula C_15_H_22_O_5_. The metabolic pathway of artemisinin primarily involves hepatic and intestinal metabolism. In hepatic metabolism, the CYP450 enzymes convert artemisinin into metabolites for easier excretion through oxidation, reduction, and hydrolysis processes (Asimus et al., 2007). Furthermore, specific bacteria in the intestinal microbiota can metabolize artemisinin *via* hydroxylation and sulphonation, these metabolites then pass into the bloodstream through the liver and kidneys before being eliminated from the body. Artemisinin has poor water and fat solubility, poor stability, and low oral bioavailability, limiting its clinical applications (Yu et al., 2012). Fortunately, several artemisinin derivatives, including dihydroartemisinin, artesunate and artemether have been synthesized (Figure 2). These derivatives are structurally slightly different but have similar therapeutic effects, anti-parasitic for instance, anti-tumor, anti-inflammatory, anti-viral, and dermatological treatments (Huang et al., 2023). Particularly, they have higher bioavailability and longer half-lives than artemisinin (Kol et al., 2022).

Artemisinin’s biological activity is linked to its peroxy bridge, derivatives are created by modifying its structure while retaining the peroxy bridge. Currently, all derivatives modify the C-9 and C-10 positions, with C-10 being the most common. Dihydroartemisinin, the simplest derivative, is obtained by reducing the carbonyl group at the C-10 position to a hydroxyl group, resulting in a molecular formula of C_15_H_24_O_5_. Studies show it has a longer half-life and higher bioavailability, but poor aqueous solubility (Zang et al., 2014; Morris et al., 2011). Artesunate, with a molecular formula of C_19_O_8_H_28_, is derived from dihydroartemisinin and succinic anhydride. It exhibits mild acidity, can penetrate biofilms, and possesses high efficacy, low toxicity, and high tolerability (Michener et al., 2023; Lin et al., 2022). Artemether is obtained by replacing the hydrogen atom on the hydroxyl group at the C-10 position with a hydrocarbon group. Compared to other derivatives, artemisinin ether derivatives are more fat-soluble but less water-soluble, with low bioavailability (de Vries and Dien, 1996).

Artemisinin and their derivatives have a wide range of pharmacologic effects, including anti-inflammatory, antioxidant, antifibrotic, and antitumor effects, in addition to their broad-spectrum antimalarial activity. These compounds have been investigated for the treatment of rheumatoid arthritis, renal injuries, gastric cancer, lung cancer, and other diseases (Yang et al., 2023). Although these studies are currently in the basic research phase, some clinical trials have shown promising results. In this review, we focused on the multistep pathogenesis of lung cancer and investigated the regulatory effect of artemisinin and its derivatives on each step of cancer progression.

## 4 Literature search

PubMed and China Knowledge Network were searched from the start date to October 2023. Search terms included “artemisinin”, “artesunate”, “dihydroartemisinin”, “artemether”, “artemisinin dimer”, “sodium artesunate”, “lung, pulmonary fibrosis”, “pulmonary nodules”, “tuberculosis”, “lung cancer”, “pneumonia”, “lung injury”, “chronic obstructive pulmonary disease”. The research papers published on the use of artemisinin and its derivatives for the treatment of lung cancer and diseases that increase the risk of cancer were included in the analysis. Finally, we studied drugs including artemisinin, dihydroartemisinin, artesunate, and artemether, the diseases including pneumonia, lung injury, lung fibrosis, and lung cancer, the details of including literature were listed at Tables 1–3. The types of research studies were animal experiments, cellular experiments, or clinical trials.

**TABLE 1:** Effects of artemisinin and its derivatives on inflammation and injury.

Disease	Animal/cell	Modeling method	Drug	Dosage	Inhibitory effect	Stimulatory effect	References
Radiation pneumonitis	Wistar rat	6MV-X line was irradiated with 15 Gy radiation dose	DHA	60 mg/kg	WBC, NF-κB, TNF-α, IL-6		Lu et al. (2022)
Influenza A virus Pneumonia	ICR mice	Influenza A virus	AS	30, 60, 120 mg/kg	TLR4, NF-κB (p65), TNF-α, IL-6, IL-1β		SUI et al. (2016)
*Pneumocystis* pneumonia	SD rats	DXM	AS	60 mg/kg	*Pneumocystis*	TLR2	Goodman et al. (2003)
*Pneumocystis* pneumonia	SD rats	DXM	DHA	60 mg/kg	*Pneumocystis*, NO, TNF-α,	CD4^+^ T cells, IFN-γ	Li et al. (2023)
*Pneumocystis* pneumonia	SD rats	DXM	Artemether	100 mg/kg	*Pneumocystis*, IL-6, IL-2		Zhu et al. (2020), YANG et al. (2021)
Hyperoxia-induced lung injury	C57BL/6 mice	Hyperoxia (75% oxygen), 14 days	AS	15 mg/kg	TNF-α, IL-6, IL-1β, NLRP3, ASC, caspase-1, MDA, p-NF-κB (p65), p-IκBα	SOD, GSH	Meng et al. (2022)
Acute lung injury	SD rats NR8383 cells	LPS	AS	7.5, 15, 25 mg/kg (*in vivo*) 5, 10, 20 μg/mL (*in vitro*)	MPO, Apoptotic cells, NLRP3, caspase-1, ASC, caspase-3	SIRT1	Wang et al. (2024b)
Acute lung injury	C57BL/6 mice	Intestinal ischemia/reperfusion	AS	—	MDA, MPO, IL-1β, TNFα, CXCL1, MCP-1, TUNEL-positive cells, Bax, caspase-3	SOD, Bcl-2, P-AKT, HO-1	Zhang et al. (2020a)
Acute lung injury	mice RAW264.7 cells	LPS	AS	5, 10, 20, 50 μg/mL (*in vitro*) 10 mg/kg (*in vivo*)	TNF-α, IL-1β, IL-6, W/D, lung injury score, MPO, inflammatory cell infiltration	GSH	Hao et al. (2022)
Acute lung injury	SD rats	LPS	AS	15 mg/kg	MPO, W/D, lung injury score, TUNEL-positive cells, cl-caspase-3	p-mTOR, p-Akt, PI3K	Zhang et al. (2023a)
Acute lung injury	C57BL/6 mice	LPS	DHA	75 mg/kg	Macrophages, Neutrophils, MPO, LDH, IL-1β, TNF-α, IL-6, ROS, MDA, W/D, p-p65, p-I-κB	SOD, GSH, Nrf2, HO-1	Zhao et al. (2017)
Acute lung injury	A549 BALB/c mice	LPS	AS	10, 20, 40 mg/kg	W/D, TNF-α, IL-1β, IL-6, TLR4, MPO, MDA, NF-κB, p-p65, p-I-κB, macrophages, neutrophils	Nrf2, HO-1	Ng et al. (2014)
Oxidative injury of the lung	BALB/c mice 16HBE	Cigarette smoke	AS	30 mg/kg	Macrophages, neutrophils, eosinophils, lymphocytes, IL-8, MDA, 3-NT, SOD	Nrf2	Holloway et al. (2018)
Suppurative lung injury	Kunming mice	Cecal ligation and puncture	AS	15 mg/kg	TNF-α, IL-6, COX-2, iNOS, NF-κB	Nrf2, HO-1	Huang et al. (2019)
Lung injury	SD rats	Paraquat	AS	—	IL-10, TNF-α, TGF-β1		Cao et al. (2016)
Lung injury	BALB/c mice BEAS-3B	Cigarette smoke	AS	10, 30, 100 mg/kg	IL-1β, MCP-1, IP-10, KC, NOX2, TNF-α, TGF-β1, MIP-2α, iNOS, MMP-9, TIMP-1, GM-CSF, 3-NT, 8-isoprostane, 8-OHdG, AKT, P-AKT, p44/42 MAPK	Nrf2, CAT	Zheng et al. (2021)
Lung injury	SD rats	Ischemia/reperfusion	AS	100 mg/kg	TNF-ɑ, IL-1β, IL-18, MPO, MDA, PERK, ATF4, CHOP, Fe^2+^	SOD	Liu et al. (2023b)
Lung injury	SD rats	hemorrhagic shock	DHA	6, 12 mg/kg	W/D, MPO, MDA, IL-12, IL-1β, TNF-α, TLR4, MyD88, p-NF-κB (p65)	SOD	Jiang et al. (2015)
Radiation-induced lung injury	C57BL/6 mice	Whole lung was irradiated with 20 Gy radiation dose	DHA	25 mg/kg	TGF-β, TNF-α, ROS, mitochondrial ultrastructure damaged	SOD	Chang et al. (2024)
Acute lung injury	white pigs	Ventricular fibrillation method	ART	4.8 mg/kg	TNF-α, IL-1β, IL-6, HMGB1, TLR4, NF-κB (p65), ELWI, PVP	OI	Xie et al. (2023)

Abbreviations: SD, Sprague Dawley, DXM, Dexamethasone; LPS, Lipopolysaccharide; DHA, Dihydroartemisinin; AS, Artesunate; ART, Artemisinin; TNF, Tumor necrosis factor; WBC, white blood cell, IL, Interleukin; TLR, Toll-like receptor; GSH, glutathione; SOD, Superoxide dismutase; MDA, Malondialdehyde; MPO, Myeloperoxidase; NLRP3, NOD-like receptor thermal protein domain associated protein 3; SIRT, Silent information regulator; W/D, Wet weight/dry weight; ELWI, Extravascular lung water index; PVB, Pulmonary vascular permeability; OI, Oxygenation index.

**TABLE 2:** Effect of artemisinin and its derivatives on precursor phase.

Disease	Animal/cell	Modeling method	Drug	Dosage	Inhibitory effect	Stimulatory effect	References
Pulmonary fibrosis	Wistar rats	Bleomycin	DHA	30, 60, 100 mg/kg	Ashcroft Score, HYP, IL-1β, IL-6, TNFα, CCL3, TGF-β1, JAK2, p-JAK2, STAT3, p-STAT3, Inflammatory cell		Michelet et al. (2021)
Pulmonary fibrosis	SD rats	Silica suspension	DHA	75 mg/kg	HYP, collagenous fiber, TGF-1, Smad2/3, Col-I,		Ji et al. (2023)
Pulmonary fibrosis	SD rats	Bleomycin	DHA	50 mg/kg	Pulmonary fibrosis, α-SMA, MDA	E-cadherin, Nrf2, HO-1, SOD, GSH	Wang et al. (2016)
Pulmonary fibrosis	SD rats Primary lung fibroblasts	Bleomycin	AS	100 mg/kg	α-SMA, collagen, Notch1, Jagged1, NICD, Hes-1		Xu et al. (2024)
Pulmonary fibrosis	SD rats Primary lung fibroblasts	Bleomycin	AS	100 mg/kg	Alveolar catarrh, Fibrosis, Ⅳ-Col, MMP-9, MMP-1, TIMP-1, TIMP-2		You et al. (2022)
Pulmonary fibrosis	SD rats	Bleomycin	DHA	25, 50, 100 mg/kg	Szapiel Score, HYP, TGF-β1, TNF-α, α-SMA, NF-κB		Liu et al. (2017)
Pulmonary fibrosis	SD rats	Bleomycin	AS	100 mg/kg	Ashcroft score, HYP, TGF-β1, Smad3, HSP47, α-SMA, Col-I		Zheng et al. (2019)
Idiopathic pulmonary fibrosis	RLE-6TN	TGF-β1	AS	2.6, 5.2, 10.4, 20.8 μmol/L	Cell proliferation, EMT, Smad3, ACTA2, vimentin	Smad7	Yang et al. (2018)
Pulmonary fibrosis	HFL-I	—	AS	1, 10, 100 mg/L	Cell cycle was arrested at the G0/G1 phase, Bcl-2, survivin, Col-III, Col-I	Apoptosis rate, Bax	Pan et al. (2021)
Pulmonary fibrosis	Human lung fibroblasts	TGF-β1	DHA	30 μm	Cell viability, Fe^2+^, FTH1, NCOA4, α-SMA	—	Deng et al. (2018)
Tuberculosis	*Mycobacterium tuberculosis*	—	ART	—	—	bactericidal effect	Wang et al. (2014)
Tuberculosis	Sprague–Dawley rats	*M*. *tuberculosis*	ART, AS	3.5 mg/kg	*Mycobacterium tuberculosis*	Bactericidal effect	Kiani et al. (2023)
Tuberculosis	*M. tuberculosis*	—	ART	—	DosRST	—	YU (2021)
Tuberculosis	ATCC35838	—	DHA	—	Destroys the bacterial cell wall	Bacteriostatic rate	Zhang (2010)
COPD	SD rats HBSMC	Cigarette smoke	AS	25, 50, 100 mg/kg	IL-6, IL-8, TNF-α, ICAM-1, ROS, α-SMA, cyclin D1, TGF-β1, Smad-2/3	GSH, PPAR-γ	Wang et al. (2015)

Abbreviations: COPD, Chronic obstructive pulmonary disease, SD, Sprague Dawley; TGF-β1, Transforming growth factor-β1; LPS, Lipopolysaccharide; DHA, Dihydroartemisinin; AS, Artesunate, ART, Artemisinin; TNF, Tumor necrosis factor; IL, Interleukin; HYP, Hydroxyproline; CCL, Chemokine (C-C motif) ligand; JAK, Janus Kinase, STAT, Signal transduction and transcriptional activator; Col, Collagen, GSH, Glutathione; SOD, Superoxide dismutase; MDA, Malondialdehyde; Nrf2, Nuclear factor erythroid2-related factor 2; HO-1, Heme Oxygenase-1; α-SMA, α-Smooth Muscle Actin; NICD, Notch intracellular domain; MMP, Matrix metalloproteinases; TIMP, Tissue inhibitor of metalloproteinase; HSP, Heat shock protein; EMT, Epithelial-mesenchymal transition; BCL, B-cell lymphoma; Bax, BCL2-Associated X; FTH1, Ferritin Heavy Chain 1; NCOA4, Nuclear receptor coactivator 4; ROS, Reactive oxygen species; PPAR, Peroxisome proliferator activated receptor.

**TABLE 3:** Effect of artemisinin and its derivatives on lung cancer.

Disease	Animal/cell	Modeling method	Drug	Dosage	Inhibitory effect	Stimulatory effect	References
Gefitinib-resistant lung adenocarcinoma	A549 cells	Gefitinib	DHA	12.5, 25, 50 μM	PARP, Bcl-2, cell viability, GSH, GPX4, FTH, p62	Caspase-3, LC3, ROS, Beclin1, Apoptosis rate	Cao et al. (2022)
NSCLC	A549, HCC827 cells BALB/c nude mice	A549 cells	DHA	0, 5, 10, 25, 50, 100 μM 10, 50 mg/kg	Cell proliferation, Bcl-2, Bcl-xL, Ki-67	Apoptosis rate, TUNEL Positive cells, PARP	Choi (2017)
Lung cancer	Lewis cells, A549 cells, C57BL mice		DHA	5, 10 mg/kg	PCNA, Ki67, GPX4	Apoptosis rate, Bax, HMGB1, MHC-I, CRT, HSP 90, COX2	Zheng and Abramovitch (2020)
NSCLC	H1975, A549, H1650, H460 cells	—	Pyronaridine	0, 5, 10, 20, 30 μmol/L	Cell proliferation, cell cycle G2 arrested, p-EGFR, p-PI3K, p-Akt, cyclin B1	Apoptosis rate, P21, PARP, JNK, DR5, caspase-3, caspase-7, caspase-8	Li et al. (2021a)
NSCLC	H1975 cells, H358 cells, C57 BL/6 mice	LLC cells	DHA, AS	AS: 0–100 μM, 25 mg/kg, DHA: 12.5 mg/kg	Growth rate, Ki-67, Glucose, ATP, lactic acid, GLUT 1, HK 2, LDHA, p-ERK, c-Myc	—	Zhang et al. (2008)
NSCLC	Lewis cells, A549 cells C57BL/6 mice	A549, LLC cells	AS	3 mg/kg	Survivin, p-AKT	c-caspase-3	Zhong et al. (2022)
lung cancer	C57BL/6 mice	LLC cells	DHA	12.5 mg/kg	CD206, Arg-1, AKT, m-TOR	CD86, iNOS, COX-2	Xing et al. (2022)
Radioresistant lung cancer	A549 cells	40 Gy of X-rays	DHA	—	Radioresistance, MLC3-II/LC3-I, CIRBP, PINK1/Parkin	—	Han et al. (2022)
Lung cancer	C57BL/6 mice	LLC cells	DHA	5, 10, 30, 60 μg/mL	Fe^2+^, GPX4, ROS/LPO, CD206	CD 86, COX-2, MDA, p53, γ-H2A.X, NF-κB, Bax, caspase-3	Han et al. (2023)
NSCLC	—	—	ART B	—	—	Connexin 43, MAPK, Fe^2+^,	Lai et al. (2023)
NSCLC	LLC,	chlorin e6	DHA	—	GPX4	ROS	Xiao et al. (2022)
NSCLC	A549, H1299 cells	—	AS	30 μg/mL	Number of invaded cells, HuR, MMP-9	—	Garg et al. (2021)
NSCLC	C57BL/6 mice	H1975, LLC cells	AS	30, 40, 60 mg/kg	TAZ, ANKRD1, PD-L1, CD274, Ki67,	CD8	Zheng et al. (2017)
NSCLC	A549 cells	—	AS, DHA	10 μM	caspase-3, β-ACTB, xCT, VDAC	TFRC	Hu et al. (2023)
NSCLC	A549, LLC cells C57BL/6 mice	LLC cells	DHA	12.5, 25, 50 mg/kg	CDK2, CDK4, Ki67, Bcl2, Bcl-xl, p-mTOR, HIF-1α, cyclin D1, cyclin E1	—	Li et al. (2022b)
Lung cancer	A549 cells, nude mice	A549 cells	AS	200 mg/kg	Cyclin B1, P34, Bcl2, p-P38, p-JNK, p-ERK	P21, P53, Bax, caspase-3, caspase-7, caspase-9	Zhang et al. (2021)
Lung cancer	A549, H1299 cells	—	AS	10 μM	NQO-1, Keap1, Nrf2	—	Yan et al. (2018)
NSCLC	H1975, A549 cells	—	AS	50 μg/mL	FN1, N-cadherin, vimentin,	E-cadherin	Zhang et al. (2022a)
Lung cancer	NCI-H23 cells, XWLC-05 cells, nude mice	NCI-H23 cells	DHA	30 mg/kg	PRIM2/SLC7A11, cell viability, β-catenin	—	Hill et al. (2021)
Lung cancer	A549 cells, BALB/c mice	A549 cells	DHA	50, 100, 200 mg/kg	CD31, NG2, HIF-1α, VEGF, MVD,	—	Yuan et al. (2020a)
Lung cancer	LLC cells, C57BL/6 mice	LLC cells	ART	50 mg/kg	LMVD, VEGF-C, p-p38	Survival rate	Hu et al. (2022)
NSCLC	A549 cells, PC-9 cells, WI-38 cells	—	DHA	0, 20, 40, 60 μM	Glucose, ATP, lactic acid, p-mTOR, p-S6, GLUT1	Apoptosis rate	Cai et al. (2021)

Abbreviations: NSCLC, Non Small Cell Lung Cancer; DHA, Dihydroartemisinin; AS, Artesunate; ART, Artemisinin; PARP, poly-ADP-ribose polymerase; ROS, Reactive Oxygen Species; GSH, Glutathione; FTH, Ferritin Heavy Chain; Bcl, B-cell lymphoma; GPX, Glutathione Peroxidase; PCNA, Proliferating Cell Nuclear Antigen; Bax, BCL2-Associated X; HMGB, High mobility group box; MHC, Major histocompatibility complex; CRT, Calreticulin; HSP, Heat shock protein; COX, Cyclooxyganese; EGFR, Epidermal Growth Factor Receptor; PI3K, Phosphatidylinositol 3-hydroxy kinase; DR, Death Receptor; JNK, c-Jun N-terminal kinase; ATP, Adenosine triphosphate; GLUT, Glucose transporter; HK, Human kallikrein; LDHA, Lactate dehydrogenase A; ERK, Extracellular signal-regulated kinase; c-Myc, Cellular-myelocytomatosis viral oncogene; AKT, Protein Kinase B; CD, Cluster of differentiation; iNOS, Inducible Nitric Oxide Synthase; mTOR, Mammalian target of rapamycin; CIRBP, Cold-inducible RNA-binding protein; MDA, Malondialdehyde; MMP, Matrix metalloproteinases; Nrf2, Nuclear factor erythroid2-related factor 2; LPO, Lipid Peroxidation; NF-κB, Nuclear factor-kappa B; MAPK, Mitogen-activated protein kinase; ANKRD, Ankyrin Repeat Domain; TFRC, Transferrin Receptor; ACTB, Actin beta; CDK, cyclin-dependent kinase; HIF, Hypoxia inducible factor; FN, Fibronectin; VEGF, Vascular Endothelial Growth Factor; MVD, Microlymphatic vessel density.

## 5 Mechanisms by which artemisinin and its derivatives ameliorate the risk of lung cancer

### 5.1 Inflammation and injury (phase Ⅰ): pneumonia and lung injury

**FIGURE 3:**
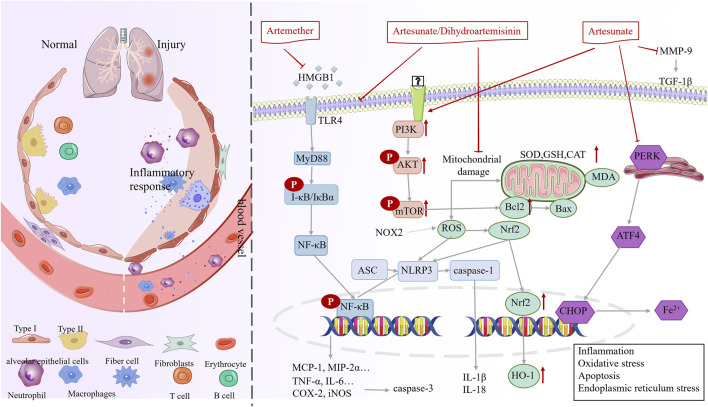
Mechanisms underlying the therapeutic effects of artemisinin in phase I (inflammation and injury).

In the inflammation and injury phase (phase Ⅰ), artemisinin and its derivatives are primarily used to alleviate pneumonia and lung injury from various causes (Table 1). The mechanism may be through modulation of immunity, antioxidant, apoptosis and endoplasmic reticulum stress (Figure 3).

#### 5.1.1 Suppression of inflammation to enhance immunomodulation

Radiation pneumonia, influenza A virus pneumonia, or *Pneumocystis carinii* pneumonia triggers the infiltration of numerous inflammatory cells, leading to lung damage (Vlahos et al., 2011; Hoving et al., 2023; Gelaw et al., 2023; Yang et al., 2022). Artemisinin and its derivatives play a crucial role in combating these diseases. Concretely, artesunate, dihydroartemisinin, and artemether decrease the expression of tumor necrosis factor-α (TNF-α), interleukin-6 (IL-6), and interleukin-2 (IL-2), increase the expression of toll-like receptor 2 (TLR2), CD4^+^ T cells, and Interferon-γ (IFN-γ), inhibit inflammation, and enhance immunity, ultimately, the number of *P. carinii* encapsulated in the lungs decreased (Liu, 2021; Zhou et al., 2008; Zhou et al., 2007a; Zhou et al., 2007b). Artesunate decreases the inflammatory response and alleviates influenza A virus pneumonia by inhibiting the toll-like receptor 4 (TLR4)/nuclear factor kappa-B (NF-κB) signaling pathway (ZHOU et al., 2022). Dihydroartemisinin inhibits the activation of the NF-κB signaling pathway, reduces the expression of inflammatory factors (TNF-α and IL-6), and decreases leukocyte counts for the treatment of radiation pneumonitis induced by A6MV-X-rays (SUI et al., 2016).

Excessive production of inflammatory factors can lead to lung damage (Goodman et al., 2003). Artesunate and dihydroartemisinin may ameliorate lung tissue inflammation through the HMGB1/TLR4/NF-κB pathway and alleviate lung injury induced by various factors, these compounds decrease inflammatory cell infiltration, immune cell (macrophages, neutrophils, eosinophils, and lymphocytes) counts, and inflammatory mediator (TNF-α, IL-1β, IL-6, IL-10, IL-12, and TGF-β) levels (Li et al., 2023; YANG et al., 2021; Luo et al., 2016; Xie et al., 2023; Jiang et al., 2015; Meng et al., 2022; Hao et al., 2022). Moreover, artesunate inhibits appendage ligation and puncture-induced NF-κB activation in lung tissue and decreases the mRNA and protein levels of COX-2 and iNOS (Cao et al., 2016).

#### 5.1.2 Antioxidant potential

Extensive results carried out show that artesunate and dihydroartemisinin ameliorate oxidative stress in hyperoxia-induced and lipopolysaccharides (LPS)-induced lung injury in a dose-dependent manner, these compounds may increase nuclearrespiratoty factor 2 (Nrf2) levels in reactive oxygen species (ROS)-sensitive cells. Further, increased Nrf2 levels promote the expression of HO-1, superoxide dismutase (SOD), and glutathione (GSH) and decrease that of malonic dialdehyde (MDA) (Chang et al., 2024), simultaneously, Nrf2 negatively regulates the activation of LPS-induced NF-κB signaling, inhibits the activity of the NF-κB pathway, and suppresses NLRP3 inflammatory vesicles (Xie et al., 2023; Huang et al., 2019; Zhao et al., 2017). These results demonstrated that the antioxidant potential of artesunate depends on the Nrf2 protein, and is closely related to the Nrf2-mediated signaling pathway (Luo et al., 2016).

Catalase is a metalloprotein oxidoreductase, which converts H_2_O_2_ into H_2_O and O_2_. A further novel finding is that artesunate promoted the activity of catalase and decreased the nicotinamide adenine dinucleotide phosphate (NADPH) protein levels, artesunate inhibited the PI3K and p42/22 MAPK signaling pathways along the way (Ng et al., 2014).

#### 5.1.3 Apoptosis inhibitor

Multiple studies have confirmed that inefficient cell burial, such as the phagocytic clearance of apoptotic cells, is a key factor in inflammation and tissue damage in conditions like pneumonia and lung injury (Zheng et al., 2021; Zhang X. et al., 2023; Wang et al., 2024b). Hence, reducing apoptosis plays a crucial role in pneumonia treatment.

Artesunate treatment decreased TUNEL-positive cell counts and apoptosis, inhibited cl-cysteinyl asparaginase-3 (cl-CASP-3) expression, and protected the cells from LPS-induced acute lung injury, notably, the effect of artesunate was attenuated when the cells were treated with a PI3K inhibitor LY294002, suggesting that the mechanism may involve the AKT/PI3K axis (Zhang E. et al., 2020). Another study suggested that artesunate ameliorated LPS-mediated release of apoptotic proteins NLRP1, CASP-3, ASC, and CASP-1 in NR8383 cells by regulating SIRT1 expression *in vivo* (Liu Z. et al., 2023). In addition, artesunate activated the AKT and HO-1 signaling pathways, decreased the expression of TUNEL and Bax proteins, and increased Bcl-2 expression to alleviate acute lung injury induced by intestinal ischemia/reperfusion in mice (Ji et al., 2023).

#### 5.1.4 Endoplasmic reticulum stress

Qian et al. reported that the protein kinase r-like endoplasmic reticulum kinase (PERK), activating transcription factor 4 (ATF4), and the C/EBP homologous protein (CHOP) expressions were increased and Fe^2+^ concentrations were elevated in rats with ischemia/reperfusion lung injury, suggesting that this injury may trigger iron-dependent cell death by activating the endoplasmic reticulum. artesunate combined with dexmedetomidine downregulated PERK, ATF4, and CHOP expression, reduced iron concentration, and attenuated iron death to ameliorate lung injury in ischemia/reperfusion rats (Xu et al., 2024).

### 5.2 Precursor phase (phase Ⅱ): PF, tuberculosis, and COPD

**FIGURE 4:**
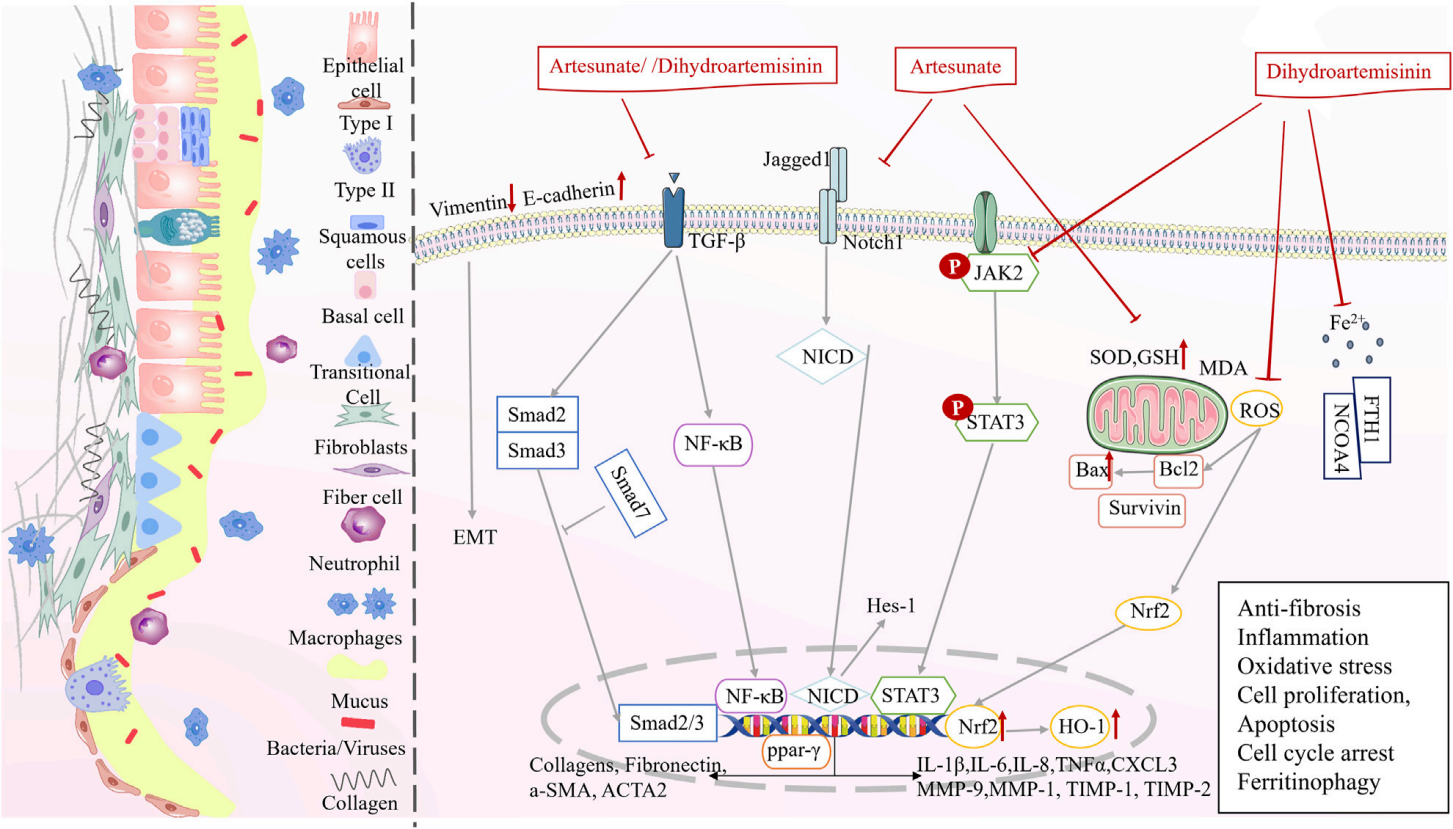
Multi-target mechanisms of artemisinin derivatives against precursor pulmonary diseases in phase II.

In precursor phase (phase Ⅱ), artemisinin derivatives exhibited anti-fibrotic, anti-inflammatory, anti-bacterial, and anti-oxidative stress to ameliorate PF, tuberculosis, and COPD (Figure 4; Table 2).

#### 5.2.1 Antifibrotic effects

Dihydroartemisinin and artesunate exhibit significant potential in combating PF. Dihydroartemisinin reduced hydroxyproline levels and decreased the Ashcroft scores in a dose-dependent manner, alleviating PF in a bleomycin induced PF model (You et al., 2022). Another study demonstrated through masson staining that dihydroartemisinin treatment decreased the number of collagen fibers in the alveolar wall and around blood vessels in PF (Zheng et al., 2019). Artesunate inhibited the Notch signaling pathway, decreased Jagged1, NICD, and Hes-1 protein expression, reduced TGF-induced α-smooth muscle actin (α-SMA) and collagen content in fibroblasts, and inhibited the differentiation of fibroblasts into myofibroblasts (Liu et al., 2017). In addition, artesunate inhibits PF by regulating the expression of the profibrotic proteins, type IV collagen, TIMP-1/2, MMP-2/9, TGF-β1, Smad3, HSP47, α-SMA, and type I collagen (Wang et al., 2016; Wang et al., 2015).

#### 5.2.2 Ameliorating inflammation and oxidative stress

Dihydroartemisinin reduces the expression of TGF-β1, a key factor in fibrosis, and inhibits the activation of JAK2 and STAT3, thereby decreasing the expression of the inflammatory factors, such as IL-1β, IL-6, TNF-α, and chemokine ligand 3, and reducing the infiltration of inflammatory cells (You et al., 2022). The Smad proteins are specific intracellular signal transduction molecules of the TGF-β1 family, and dihydroartemisinin inhibits the expression of Smad2/3 in the rat serum (Zheng et al., 2019). Dihydroartemisinin treatment markedly reduces the number of neutrophils and macrophages and decreases the expression of inflammatory stressors in the lung tissue of PF rats, which may be mediated by the inhibition of the NF-κB signaling (Deng et al., 2018). In addition, dihydroartemisinin regulates the oxidative stress through the Nrf2/HO-1 signaling pathway, leading to a decrease in MDA and α-SMA levels and an increase in SOD, GSH, and E-cadherin levels (Yang et al., 2018). Artesunate treatment of COPD rats reduces IL-6, IL-8, TNF-α, and ICAM-1 expression, reverses smoking-induced increase in ROS levels and reduction in GSH levels, and attenuates inflammatory infiltration and oxidative stress (Pan et al., 2021). From these results it is clear that dihydroartemisinin and artesunate can ameliorate inflammation and oxidative stress.

#### 5.2.3 Inhibits cell proliferation and promotes apoptosis

Recent studies indicate that artesunate offers benefits in enhancing the cell cycle, suppressing hyperproliferation, and stimulating apoptosis. Artesunate inhibits TGF-β1-induced epithelial-to-mesenchymal differentiation (EMT) and RLE-6TN cell proliferation by upregulating Smad7 mRNA and protein expressions and downregulating Smad3, ACTA2, and Vim mRNA expressions (Wang et al., 2014). Artesunate inhibited the proliferation of HFL-I cells in a time- and concentration-dependent manner, and apoptotic cells were seen after staining with Hochst33258. The progression of HFL-I cells from the G1 to the S phase was blocked, resulting in a build-up of cells in the G1 phase (which were unable to enter the S phase), a relative increase in the number of cells in the G2/M phase, a decrease in the expression of Bcl-2 and survivin mRNA, an increase in the expression of Bax mRNA, and an increase in the number of apoptotic cells (Zhang, 2010). In addition, artesunate decreased α-SMA and cell cycle protein D1 levels and inhibited cell proliferation by targeting the PPAR-γ/TGF-β1/Smad2/3 signaling pathway (Pan et al., 2021).

#### 5.2.4 Promotes iron-mediated autophagy

Wang et al. used dihydroartemisinin to intervene in a fibrotic cell model established using a human embryonic lung fibroblast cell line and found that dihydroartemisinin inhibited cell viability, decreased Fe^2+^ levels, and inhibited the expression of ferritin heavy chain 1 (FTH1) and nuclear receptor coactivator 4 (NCOA4) genes and proteins. Specifically, dihydroartemisinin reduced Fe^2+^ levels at an early stage and triggered iron autophagy, resulting in the degradation of iron autophagy-related proteins, FTH1 and NCOA4, followed by an increase in Fe^2+^ levels (YU, 2021). Thus, dihydroartemisinin may have a dual role in inhibiting oxidative stress at the early stage and promoting iron-autophagy in the later stage.

#### 5.2.5 Antimycobacterial effects

*Artemisia annua*, artemisinin, and their derivatives have a bacteriostatic effect on *M. tuberculosis* (Kiani et al., 2023; Gu et al., 2021; Zheng et al., 2017; Choi, 2017). Dihydroartemisinin co-administration mitigates rifampicin resistance, disrupts *Mycobacterium bovis* cell wall integrity, and promotes the inhibition of mycobacteria (Gu et al., 2021). DosRST is a two-component regulatory pathway induced by host immune signals (e.g. hypoxia, nitric oxide, and carbon monoxide), and inhibition of DosRST decreases the reservoirs of persistent drug-resistant bacteria in the host (Zheng and Abramovitch, 2020). Artemisinin inhibits the DosRST signaling and thus reduces the *M. tuberculosis* (Zheng et al., 2017).

### 5.3 Cancer phase (phase Ⅲ): lung cancer

Artemisinin and its derivatives, alone or in combination, can be effective in controlling precancerous diseases and lung cancer development and progression. Despite the limited number of clinical studies, the anti-lung cancer efficacy of artemisinin and its derivatives shows promise. The results of three lung cancer-related clinical trials showed that artesunate combined with chemotherapy upregulated the CD39, CD279, and GrzB expression in CD8^+^ and CD4^+^ T cells in patients with lung cancer, thereby modulating the immune function of T-cell subsets. This improved the disease control rate and near-term survival and prolonged the time to progression of disease in patients with advanced NSCLC without an increase in adverse reactions (Xing et al., 2022; Zhang et al., 2008).

**FIGURE 5:**
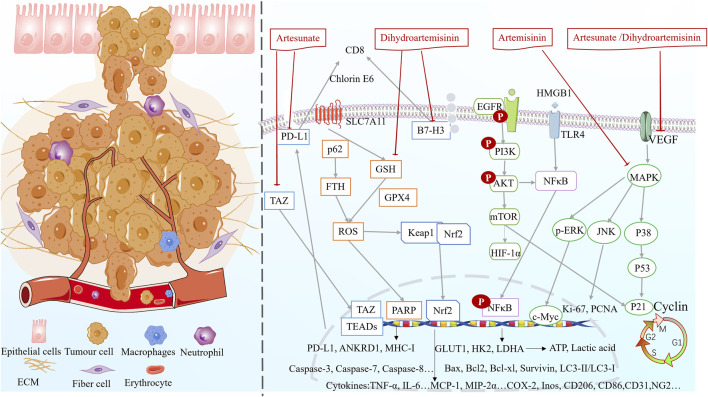
Multifunctional mechanisms of artemisinin and its derivatives against lung cancer.

Fundamental research indicated that artemisinin, dihydroartemisinin and artesunate can regulate inflammation, oxidative stress, glycolysis, ferroptosis, inhibit cell proliferation, promote apoptosis (Figure 5), alleviate lung cancer symptoms and reduce drug resistance (Table 3). However, in the current experimental reports, due to the large heterogeneity between experiments, it is not possible to obtain the differences in drug responses of artemisinin and its derivatives in treating different subtypes of lung cancer (such as EGFR mutations and KRAS mutations) (Cai et al., 2021; Yan et al., 2018; Cao et al., 2022). More subtype-specific preclinical trials are expected to further explore the drug advantages.

#### 5.3.1 Suppressing inflammation and enhancing immunomodulation

Artesunate inhibits NSCLC cell growth by inhibiting the TAZ/PD-L1 signaling and increasing CD8^+^ T cell infiltration (Cao et al., 2022). Dihydroartemisinin downregulates B7-H3 but not PD-L1 expression on NSCLC cells. Notably, B7-H3 participates in dihydroartemisinin-mediated antitumor effects by increasing intratumoral CD8^+^ T lymphocyte counts in NSCLC (Hu et al., 2023). Han et al. found that dihydroartemisinin promoted immunogenic death in lung cancer mice, increased the expression of related proteins (MHC-I, CRT, and HSP90), and upregulated HMGB1 expression (Han et al., 2023). In addition, dihydroartemisinin increased the expression of M1 phenotype-related molecules (CD86, iNOS, and Cox-2) and decreased that of M2 phenotype-related molecules (CD206 and Arg-1). Therefore, dihydroartemisinin promoted the macrophage M0/M1 phenotypic shift and acted as an immunomodulator for macrophage M2 to M1 reprogramming (possibly by regulating the AKT/mTOR pathway) (Xiao et al., 2022).

#### 5.3.2 Antioxidant effects

Artesunate activates the protective Keap1/Nrf2 pathway in lung cancer cells, improving cellular antioxidant defenses (Hill et al., 2021). Dihydroartemisinin treatment results in high levels of ROS and significantly inhibits A549 cell proliferation (Lai et al., 2023). Artesunate and dihydroartemisinin reduce the voltage-dependent anion channel 1 protein levels and cleavage CASP-3, possibly mediating mitochondrial disruption through ROS (Zhang et al., 2021).

#### 5.3.3 Inhibition of cell proliferation and promotion of apoptosis

Dihydroartemisinin inhibited the proliferation of A549/HCC 827 cell lines in a dose-dependent manner, and HCC 827 cells were more sensitive to dihydroartemisinin inhibition compared with A549 cells (Hu et al., 2023). Dihydroartemisinin significantly downregulates NSCLC proliferation-associated factors (Ki-67 and PCNA) and increases the percentage of TUNEL-positive cells (Hu et al., 2023). Artesunate inhibits the proliferation of A549 cells and reduces the number of positive cells. Notably, it is more effective in combination with cisplatin (Li W. et al., 2021).

CASP-3 is upregulated after dihydroartemisinin intervention in A549-GR cells, whereas PARP and Bcl-2 are downregulated, alleviating gefitinib resistance and increasing apoptosis (Lai et al., 2023). In addition, the expression of the apoptotic protein Bax was synchronously enhanced (Han et al., 2023). Pyrrolidine (artemisinin synthetic drug) can upregulate DR5 expression by activating JNK, triggering the TRAIL-induced apoptosis pathway, and upregulating the expression of PARP, caspase-3, caspase-7, and caspase-8 (Zhong et al., 2022). Artesunate promotes apoptosis in A549 cells by inhibiting the AKT/survivin signaling (Zhang W. et al., 2022). Artesunate combined with cisplatin induces morphologic changes, such as cellular crumpling, nuclear chromatin condensation, and irregular shape. The combined treatment downregulates the activity of the anti-apoptotic molecule Bcl-2, upregulates the expression of the pro-apoptotic molecules P53 and Bax, and increases the activity of caspases to promote endogenous apoptosis. Notably, the synergistic effect of the combination therapy may be mediated by the P38/JNK/ERK MAPK pathway (Li W. et al., 2021).

#### 5.3.4 Ferroptosis induction

Dihydroartemisinin inhibits the downregulation of the expression of the ferroptosis-related proteins GPX4 and FTH in gefitinib-resistant A549 cells, thereby contributing to free iron release (Lai et al., 2023). Dihydroartemisinin-triggered iron death of tumor-associated macrophages releases ROS/LPO, inducing the expression of COX-2 and accumulation of MDA. Consequently, DNA damage occurs, which activates downstream NF-κB to remodel tumor-associated macrophages to the M1 phenotype (Li LG. et al., 2022). Dihydroartemisinin inhibits GPX4 and enhances ROS production to promote the therapeutic effects of chlorin e6-induced photodynamic therapy in lung cancer (Han et al., 2022). Ferritin-1, a ferroptosis inhibitor, restores dihydroartemisinin-induced decrease in cell viability and cell death in NCI-H23 and XWLC-05 cells. Notably, dihydroartemisinin inhibits proliferation and colony formation and induces ferroptosis in lung cancer cells by inhibiting the PRIM2/SLC7A11 axis (Yuan B. et al., 2020).

#### 5.3.5 Inhibition of cell invasion and migration

Artesunate inhibits the proliferation, migration, and invasion of A549 and H1299 cells and induces their apoptosis, possibly due to reduced HuR and MMP-9 protein expressions (Hu et al., 2022). Artesunate treatment downregulates the transcription of BTBD549 and increases the levels of epithelial cell markers (E-calmodulin), whereas the levels of mesenchymal cell markers (including N-calmodulin, vimentin, and FN1) are significantly reduced. Artesunate suppresses EMT in a dose-dependent manner, thereby suppressing the migratory capacity of NSCLC cells (Wang et al., 2020).

#### 5.3.6 Cell cycle blockade

Pyrrolidine inhibits the EGFR/PI3K/Akt signaling pathway, increases P21 expression, decreases cyclin B1 expression, and inhibits EGFR-dependent NSCLC cell growth and cell cycle blockade in the G2 phase (Zhong et al., 2022). Artesunate blocks the cell cycle in the G0/G1 phase in both H1975 and LLC cells and induces G2/M cell cycle blockade in H460 cells (Cao et al., 2022). The combination of artesunate and cisplatin significantly enhances cell cycle arrest in the G2/M phase, upregulates P21 expression, and downregulates cyclin B1 and P34 expression (Li W. et al., 2021). Dihydroartemisinin can induce A549 cell cycle arrest by reducing the expression levels of key G0/G1 regulators, including cyclin dependent kinase 2 (CDK2), cyclin dependent kinase 4 (CDK4), and cyclin E1, and the mTOR/HIF-1α signaling is one of the potential key pathways involved (Li Y. et al., 2021).

#### 5.3.7 Autophagy inducing effect

Autophagy was significantly upregulated in A549-GR cells after dihydroartemisinin treatment, and the expression of LC3 and Beclin1 (autophagy-related proteins) increased, while p62 decreased (Lai et al., 2023). Dihydroartemisinin reduced LC3-II/LC3-I expression, inhibited mitochondrial autophagy, and ameliorated radioresistance in the lung cancer A549 cell line (A549R), the key target of which may be cold-inducible RNA-binding protein (Wu et al., 2022).

#### 5.3.8 Inhibition of angiogenesis

Dihydroartemisinin inhibited tumor vascularization by decreasing the expression of HIF-1α, VEGF, and endothelial cell-specific marker (CD31 and NG2) proteins, leading to a significant reduction in microvessel and mature vessel density in a time-dependent manner (Zhang et al., 2013). Artemisinin significantly reduced p38 MAPK phosphorylation of VEGF-C in a dose-dependent manner and had a significant inhibitory effect on tumor lymphatic microvessel density in the peritumor area, ultimately increasing the survival of lung cancer mice (Wang et al., 2008).

#### 5.3.9 Regulation of glycolysis

Artesunate and dihydroartemisinin downregulate the extracellular signal-regulated kinase activity, inhibit c-Myc expression, and decrease glucose transporter protein (GLUT1), human myosin-releasing enzyme (HK 2), and lactate dehydrogenase A concentrations in a dose-dependent manner. Consequently, glucose uptake, ATP content, and lactate production decrease in NSCLC cells, leading to the inhibition of aerobic glycolysis *in vitro* and *in vivo* (Zhang Y. et al., 2022). Dihydroartemisinin-induced inhibition of GLUT1 inhibits mTOR, decreasing glucose uptake and glycolytic metabolism in NSCLC cells (Mi et al., 2015).

Artemisinin and its derivatives have therapeutic effects on asthma, respiratory distress syndrome, novel coronavirus pneumonia, pulmonary hypertension, and silicosis (Zhang M. et al., 2023; Zhou et al., 2021; Cai et al., 2022; Xin et al., 1998; Cui et al., 2022). Overall, artemisinin and its derivatives are promising therapeutic agents for lung diseases. Subsequent studies should focus on the therapeutic effects of artemisinin and its derivatives in other lung diseases in addition to their effects in pre-cancerous lung diseases. In addition to artesunate and dihydroartemisinin, other derivatives, such as artemisinin dimer and artesunate sodium, can also be evaluated for their role in the prevention and treatment of Lung cancer.

## 6 Mechanism of artemisinin and its derivatives in the multistep dynamic development process of lung cancer

High mobility group box 1 protein (HMGB1) is an upstream signaling protein that regulates inflammation and activates TLR4. TLRs are a family of innate immune recognition receptors that activate myeloid differentiation factor 88 and NF-κB (Yuan et al., 2023), NF-κB is a transcription factor that plays a key role in cellular inflammatory and immune responses (Yuan J. et al., 2020). Artesunate and dihydroartemisinin may exert immunomodulatory effects by regulating HMGB1 expression, inhibiting TLR4/NF-κB activation, decreasing TNF-α, IL-6, IL-1β, iNOS, and Cox-2 expression, and attenuating inflammation caused by pneumonia, lung injury, lung fibrosis, and lung cancer (Deng et al., 2018; Han et al., 2023).

TGF-β1 is a cytokine that regulates cell growth and differentiation and promotes the transformation of lung fibroblasts into myofibroblasts, which then synthesize and release high concentrations of matrix proteins components into the extracellular matrix, leading to lung fibrosis. The Smad proteins are specific intracellular signaling molecules of the TGF family. TGF-β1 signaling induces the phosphorylation of JAK2, which activates the JAK2/STAT3 signaling pathway to promote inflammatory response and fibrosis. Dihydroartemisinin reduces the expression of TGF-β1 and, Smad2/3, inhibits the activation of JAK2/STAT3, attenuates the expression of inflammatory factors (IL-6, TNFα, and chemokine ligand 3), and reduces the infiltration of inflammatory cells (You et al., 2022; Zheng et al., 2019). In addition, dihydroartemisinin can inhibit PD-L1 expression, promote T-cell growth, and increase the killing capacity of T cells. Ultimately, dihydroartemisinin prevents tumor immune escape by inhibiting the TGF-β, PI3K/Akt, and STAT3 signaling pathways to promote tumor eradication (Zhang E. et al., 2020; Zhang H. et al., 2020).

Nrf2 is an important initiator of the oxidative stress pathway. The activated Nrf2 translocates to the nucleus and regulates the transcription of antioxidant proteins such as HO-1. ROS are mainly generated by redox reactions and have a dual role in tumor cells. SOD, GSH, and MDA are the common biomarkers of oxidative stress. Artesunate and dihydroartemisinin reduced oxidative stress in lung tissues in a dose-dependent manner by modulating the Keap1/Nrf2 signaling pathway. Notably, Nrf2 translocates to the nucleus in ROS-sensitive cells, increases the antioxidant HO-1 levels, decreases the MDA levels, and increases the SOD and GSH activities (Xie et al., 2023; Huang et al., 2019; Zhao et al., 2017; Xin et al., 1998). In addition, artesunate can enhance the antioxidant defense system and prevent oxidative damage in the lungs by inhibiting the PI3K and p42/22 MAPK signaling pathways, decreasing the levels of oxidative biomarkers (8-IPS, 8-OHdG, and 3-NT), promoting anti-hydrogen peroxide dismutase activity in lung tissues, and decreasing the expression of NADPH (Ng et al., 2014).

The combination of artesunate and dihydroartemisinin downregulates PERK, ATF4, and CHOP, reduces Fe^2+^ concentration, attenuates iron-induced cell death, and ameliorates lung injury (Xu et al., 2024). Dihydroartemisinin decreases GPX4, FTH1, and NCOA4 expression and reduces Fe^2+^ levels by inhibiting the PRIM2/SLC7A11 axis (YU, 2021; Lai et al., 2023; Yuan B. et al., 2020).

Artesunate may inhibit the proliferation of lung cancer cells, downregulate the expression of anti-apoptotic molecules Bcl-2 and survivin, upregulate the expression of pro-apoptotic molecules P53 and Bax, and increase the activity of caspases and apoptosis rate through the PPAR-γ/TGF-β1/Smad2/3, AKT/Survivin, P38/JNK/ERK, and MAPK pathways (Pan et al., 2021; Wang et al., 2014; Zhang, 2010; Li W. et al., 2021; Xin et al., 1998). This compound increases the expression of E-calmodulin, decreases the levels of N-calmodulin, vimentin, and FN1, inhibits EMT, and decreases the migratory ability of NSCLC cells (Wang et al., 2020). Moreover, artesunate induces the G2/M cell cycle blockade in HFL-I and H460 cells and blocks the cell cycle in the G0/G1 phase in H1975 and LLC cells. Notably, the combination of artesunate with cisplatin enhanced cell cycle blockade in the G2/M phase (Zhang, 2010; Cao et al., 2022; Li W. et al., 2021).

Dihydroartemisinin inhibited the proliferation of A549 and HCC827 cells in a dose-dependent manner. In addition, dihydroartemisinin treatment significantly downregulated Ki-67, PCNA, PARP, and Bcl-2 expressions, upregulated cysteine 3 and Bax expressions, and increased the percentage of TUNEL-positive cells. Moreover, dihydroartemisinin may decrease the expression levels of the key G0/G1 regulators, CDK2/4, and cyclin E1 through the mTOR/HIF-1α signaling, thereby inducing A549 cell cycle blockade and alleviating gefitinib resistance (Hu et al., 2023; Lai et al., 2023; Li Y. et al., 2021). Overall, artemisinin and dihydroartemisinin regulate multiple pathways, such as TLR4/NF-κB, Keap1/Nrf2, mTOR/HIF-1α, PI3K/Akt, AKT/mTOR, JAK2/STAT3, and MAPK, by modulating cellular processes including inflammation, immunity, oxidative stress, ferroptosis, apoptosis, cell proliferation, and cell cycle arrest. Thereby, they ameliorate lung cancer precursor lesions such as pneumonia, lung injury, PF, and COPD, consequently reducing the risk of cancer. In addition, artemisinin and its derivatives regulate several cellular processes, including glycolysis, angiogenesis, and cellular autophagy, even when lung injury, PF, and COPD are not involved. These research directions may be explored in the future.

## 7 Adverse effects of artemisinin and its derivatives and current management approaches

### 7.1 Adverse effects and safety of drugs

Artemisinin and its derivatives have minimal adverse reactions and side effects (Lee et al., 2010; Asghari et al., 2015). Trendfilova et al. reported that artemisinin and its derivatives are unlikely to cause adverse effects in humans, probably due to the low clinical doses and the short duration of administration (Trendafilova et al., 2020). Whereas, several studies have demonstrated that high-dose and long-term administration of artemisinin-based drugs has gastrointestinal, neurotoxic, and cardiotoxic effects in experimental animals (e.g., rhesus monkeys, rats, and dogs), and the most common adverse effects are nausea, vomiting, and dizziness (Li et al., 2019; Li X. et al., 2022).

More specifically, oral administration is safer than intramuscular injection in animal models because artemisinin is present in experimental animals for a long period after its slow release from intramuscular formulations, leading to severe side effects (Gordi and Lepist, 2004). Intramuscular injection of artemether was more neurotoxic than that of artesunate in a mouse model, suggesting differences in the optimal dosing of different derivatives (Nontprasert et al., 1998; Efferth and Kaina, 2010). Therefore, artemisinin and its derivatives need to be extensively tested in clinical trials for selecting drugs, dosage regimens, duration of therapy, and route of administration for different lung diseases.

### 7.2 Current management approaches: to optimize drug delivery systems

Despite the promising pharmacologic effects of artemisinin and its derivatives, their clinical applications are limited due to their poor aqueous solubility, short half-life in blood circulation, low bioavailability, and poor stability (Alven and Aderibigbe, 2020; Qian et al., 2021). In recent years, several micro/nanoscale delivery systems, such as polymer-drug nanoparticles, micelles, lipid nanoparticles, and liposomes have been developed to improve the therapeutic efficacy and reduce the adverse effects of these compounds in lung cancer and precancerous diseases (Abdelaziz et al., 2018; Kim et al., 2023).

#### 7.2.1 Polymer-drug nanoparticles

The protective coating of polyethylene glycol (PEG) inhibits the detection and clearance of nanoparticles by the immune system and prolongs drug circulation time (Steffes et al., 2020). Hao et al. synthesized PEGylated artesunate precursor drug (mPEG-ART) and found that the precursor drug ameliorated LPS-induced acute lung injury, suggesting its potential use as an anti-inflammatory agent (Hao et al., 2022).

Dai et al. linked dihydroartemisinin to a multi-armed PEG, and this coupling increased the dihydroartemisinin loading capacity, enhanced the water solubility, and increased the half-life of the drug in blood circulation, resulting in better inhibition of tumor growth (Dai et al., 2014). Kumar et al. synthesized a new hyaluronic acid–dihydroartemisinin conjugate in which the hydroxyl group of dihydroartemisinin was covalently linked to the carboxyl group of hyaluronic acid to increase the drug loading capacity by 12% and improve the therapeutic efficacy (Kumar et al., 2019). Sun et al. encapsulated dihydroartemisinin in gelatin or hyaluronic acid nanoparticles using an electrostatic field system to form polymers of approximately 30–40 nm diameter, and the encapsulation efficiencies were 13% and 35% with gelatin and hyaluronic acid, respectively, which improved the bioavailability of dihydroartemisinin (Sun et al., 2014).

#### 7.2.2 Lipid nanoparticles

Folic acid-modified PEGylated paclitaxel and artemether solid lipid nanoparticles (SLNs) were prepared using a high-pressure homogenization technique. SLNs showed enhanced cytotoxicity and increased relative drug bioavailability. Pharmacodynamic studies confirmed the enhanced anticancer potential of the SLN formulations without any hepatic or renal toxicity (Khatri et al., 2020). Chen et al. used the ROS-responsive fraction of thioacetal to bridge cinnamaldehyde and dihydroartemisinin. The precursor drug combined with photodynamic therapy enhanced the antitumor effect of dihydroartemisinin by laser irradiation-induced ROS degradation in cancer cells (Chen et al., 2022).

#### 7.2.3 Liposomes

Liposomes are the biocompatible, degradable, non-toxic, and non-immunogenic structures prepared from phospholipids and cholesterol (Najlah et al., 2019). Fu et al. constructed a biomineralized liposome (LDM) by incorporating dihydroartemisinin into the liposome core and encapsulating pH-responsive calcium phosphate on the liposome surface as a shell (Fu et al., 2023). Drug delivery to the lungs through nebulization resulted in approximately 6.80-fold higher drug accumulation in lung lesions compared with the delivery through intravenous injection. Degradation of the shell induced Ca^2+^ burst to create a “Ca^2+^ burst–endoplasmic reticulum stress–iron apoptosis” cycle, enhancing iron apoptosis in lung cancer cells. Consequently, LDM promoted tumor elimination *in vitro* and *in vivo*. Hu et al. prepared liposomes of artesunate using the film dispersion method and lyophilized the preparation to obtain liposomal artesunate dry powder inhalers, which showed potent anti-inflammatory effects in acute lung injury treatment (Hu et al., 2016). In addition, the liposomal formulation improved the bioavailability of artesunate and dihydroartemisinin in the lungs and increased the therapeutic efficacy of drugs.

#### 7.2.4 Other drug delivery systems

Non-ionic surfactant vesicles (Niosomes) are a new type of nanocarriers optimal for encapsulating lipophilic and hydrophilic drugs (Gharbavi et al., 2018). Shahbazi et al. prepared artemisinin and metformin (ART + MET)-loaded PEGylated niosomes in different dosages using the thin film hydration method and found that these niosomes had higher antiproliferative effects on A549 lung cancer cells compared with free ART-MET (Shahbazi et al., 2023). ART-loaded porous polylactic acid-hydroxyacetic acid copolymer microspheres were prepared using the emulsification solvent volatilization method. The microsphere-released drug was effectively taken up by A549 cells and had a strong inhibitory effect on cell migration and invasion by inducing apoptosis and cell cycle arrest in the G2/M phase (Xiong et al., 2021).

#### 7.2.5 Potential for drug modification

The dimers of artemisinin and the development of hybrid drugs have shown significant potential. Dimers enhance activity by linking two molecules of artemisinin, such as a 2-5-fold increase in anti-malarial activity and stronger inhibition of the PI3K/Akt pathway, which can overcome tumor resistance (Çapcı et al., 2021; Yue et al., 2023; Chen et al., 2025; Jiang et al., 2025). Hybrid drugs integrate heterogeneous active units; for example, artemisinin-indirubin hybrids can fight cancer through dual pathways, with an efficacy increase of more than threefold. These modifications break through the limitations of single-target approaches, have the advantage of multi-pathway intervention, and can also optimize toxicological properties through structural design (Xu et al., 2023; Wang et al., 2023b; Wang P. et al., 2023; Wang et al., 2023d). However, controllable synthesis, compatibility with delivery systems, and insufficient clinical evidence are current challenges. It is necessary to combine computational design with intelligent delivery to promote transformation and provide new pathways for the treatment of pulmonary diseases.

### 7.3 Future possibilities and current limitations

Artemisinin and its derivatives have demonstrated remarkable multi - stage intervention characteristics and hold significant potential for clinical translation. In particular, their dual role in preventing the progression of lung injury - fibrosis - cancer and enhancing chemotherapy sensitivity will make a substantial contribution to the future development of medicine. As a paradigm for the development of natural product drugs, artemisinin compounds offer new ideas for overcoming clinical challenges such as drug resistance in current targeted therapies, and their broad - spectrum biological activity based on the peroxide bridge structure is expected to break through the limitations of traditional single - target drugs.

However, there are still multiple challenges in moving from basic research to clinical application. First, the safety profiles under different formulations (such as nanoformulations vs traditional formulations), routes of administration (inhalation vs intravenous), and dose gradients have not been fully clarified, especially the need for systematic evaluation of neurotoxicity and immune regulation effects with long - term use. Second, although there is abundant evidence from basic research, there is still a significant evidence gap in clinical trials targeting populations, lacking large sample size data to support efficacy and safety. In addition, like all natural products, efficient targeted delivery of artemisinin compounds remains a key bottleneck restricting clinical translation, and their pharmacokinetic defects such as poor water solubility and short plasma half - life urgently need to be optimized.

## 8 Summary

This review highlighted the fact that the dynamic development processes of lung diseases to lung cancer, elaborated on the pathologic states of pre-lung cancer diseases and the mechanisms by which they progress to lung cancer. In addition, we discussed the therapeutic effect of artemisinin and its derivatives on different diseases that increase the risk of lung cancer and explored the common regulatory mechanisms. Finally, we summarized the development of targeted drug delivery systems for artemisinin and its derivatives.

Pneumonia, lung injury, PF, tuberculosis, and COPD increase the risk of lung cancer to varying degrees. Artemisinin and its derivatives can reduce DNA damage, oxidative stress, and inflammation, inhibit cell proliferation, promote apoptosis, and regulate the cell cycle through multiple pathways, such as the TLR/NF-κB, Keap1/Nrf2, and PI3K/Akt signaling pathways, thereby exerting a therapeutic effect on lung cancer and pre-lung cancer diseases. Moreover, these compounds can regulate glycolysis, inhibit angiogenesis, increase cellular autophagy, and repair lung injury. Nanoscale delivery systems, such as polymer-drug nanoparticles, micelles, and liposomes, are being developed to increase their bioavailability and improve drug stability, which will improve the therapeutic efficacy.

Artemisinin and its derivatives can be used as both anti-lung cancer and lung protective agents in clinical application. Current research focuses on artesunate and dihydroartemisinin. However, clinical trials to verify their efficacy are still lacking. Future studies should focus on more artemisinin derivatives, and clinical trials should be conducted to validate the efficacy of artemisinin-based approaches for the prevention and treatment of lung cancer.
